# A novel short L-arginine responsive protein-coding gene (*laoB*) antiparallel overlapping to a CadC-like transcriptional regulator in *Escherichia coli* O157:H7 Sakai originated by overprinting

**DOI:** 10.1186/s12862-018-1134-0

**Published:** 2018-02-12

**Authors:** Sarah M. Hücker, Sonja Vanderhaeghen, Isabel Abellan-Schneyder, Romy Wecko, Svenja Simon, Siegfried Scherer, Klaus Neuhaus

**Affiliations:** 1 Chair for Microbial Ecology, Wissenschaftszentrum Weihenstephan, Technische Universität München, Weihenstephaner Berg 3, 85354 Freising, Germany; 2 Department of Computer and Information Science, University of Konstanz, Box 78, 78457 Konstanz, Germany; 3 ZIEL – Institute for Food & Health, Technische Universität München, Weihenstephaner Berg 3, 85354 Freising, Germany; 4 Core Facility Microbiome/NGS, ZIEL – Institute for Food & Health, Technische Universität München, Weihenstephaner Berg 3, 85354 Freising, Germany; 5 Fraunhofer ITEM-R, Am Biopark 9, 93053 Regensburg, Germany

**Keywords:** Overlapping gene, Overprinting, Small protein, De novo gene, EHEC

## Abstract

**Background:**

Due to the DNA triplet code, it is possible that the sequences of two or more protein-coding genes overlap to a large degree. However, such non-trivial overlaps are usually excluded by genome annotation pipelines and, thus, only a few overlapping gene pairs have been described in bacteria. In contrast, transcriptome and translatome sequencing reveals many signals originated from the antisense strand of annotated genes, of which we analyzed an example gene pair in more detail.

**Results:**

A small open reading frame of *Escherichia coli* O157:H7 strain Sakai (EHEC), designated *laoB* (L-arginine responsive overlapping gene), is embedded in reading frame −2 in the antisense strand of ECs5115, encoding a CadC-like transcriptional regulator. This overlapping gene shows evidence of transcription and translation in Luria-Bertani (LB) and brain-heart infusion (BHI) medium based on RNA sequencing (RNAseq) and ribosomal-footprint sequencing (RIBOseq). The transcriptional start site is 289 base pairs (bp) upstream of the start codon and transcription termination is 155 bp downstream of the stop codon. Overexpression of LaoB fused to an enhanced green fluorescent protein (EGFP) reporter was possible. The sequence upstream of the transcriptional start site displayed strong promoter activity under different conditions, whereas promoter activity was significantly decreased in the presence of L-arginine. A strand-specific translationally arrested mutant of *laoB* provided a significant growth advantage in competitive growth experiments in the presence of L-arginine compared to the wild type, which returned to wild type level after complementation of *laoB in trans*. A phylostratigraphic analysis indicated that the novel gene is restricted to the *Escherichia/Shigella* clade and might have originated recently by overprinting leading to the expression of part of the antisense strand of ECs5115.

**Conclusions:**

Here, we present evidence of a novel small protein-coding gene *laoB* encoded in the antisense frame −2 of the annotated gene ECs5115. Clearly, *laoB* is evolutionarily young and it originated in the *Escherichia/Shigella* clade by overprinting, a process which may cause the de novo evolution of bacterial genes like *laoB*.

**Electronic supplementary material:**

The online version of this article (10.1186/s12862-018-1134-0) contains supplementary material, which is available to authorized users.

## Background

The DNA triplet code is constructed such that the majority of amino acids (AA) can be encoded by more than one codon, leading to the so-called degeneration of the genetic code. Codon position three shows the highest degeneration (wobble position), whereas position one is only slightly degenerated and position two is not degenerated [1]. Thus, a DNA double strand contains six possible reading frames, each of which has the capacity to encode a protein and it is feasible that the sequences of two or more protein-coding genes overlap. Most generally, overlapping genes (OLGs) share at least one nucleotide between the coding regions of two genes. When the reading frame of the evolutionary older established gene (mother gene) is defined as frame +1, a same-strand overlap in reading frames +2 or +3 relative to the annotated gene is possible. Same-strand OLGs originate from programmed ribosomal frameshift [2, 3] or programmed transcriptional realignment [4]. Additionally, a second gene can overlap the mother gene antisense in frames −1, −2 or −3. It is under debate, which antisense frame is preferred for the occurrence of OLGs. In *E. coli,* most long antisense open reading frames (ORFs) are detected in frame −1 [5]. However, this finding might be caused by codon bias of the mother gene [6, 7]. Whereas Krakauer [1] predicted highest constraints on frame −2, in *E. coli* more long ORF are found in frame −2 than in −3 [6, 8]. Lèbre and Gascuel [9] investigated the constraints of OLGs at the AA level and detected the highest constraints on frame −3 due to a high number of “forbidden dipeptides” within the protein encoded, which would cause a stop codon in the established gene.

In prokaryotes, many genes are organized in operons, which are transcribed as a polycistronic mRNA. In these cases, trivial same-strand overlaps of only a few base pairs are very common and facilitate translational coupling [10]. In contrast, almost no long OLGs (overlap ≥90 bp) have been described in bacteria [11–14], while longer OLGs are well known in viral genomes, probably leading to genome size reduction, since in viruses, 38% of all AA are encoded overlapping and in many cases the OLGs encode accessory proteins with unusual sequence composition like many disordered regions [15–17].

OLGs may originate by overprinting [18]: By chance, an overlapping reading frame is expressed in a bacterial population. However, encoding two functional genes at one locus leads to severe constraints of sequence evolution, since many mutations will influence the AA sequence of two genes carrying completely different functions [1, 8, 9]. This may be one reason why the overprinting hypothesis has been neglected as being rather unlikely [7, 19]. Instead, the gene duplication followed by subfunctionalization or neofunctionalization hypothesis [20] has been favored for the origin of novel genes.

Here, we present an initial functional characterization of the novel OLG *laoB* of EHEC, the expression of which was seen in transcriptome data and ribosomal profiling [21]. *laoB* overlaps antiparallel to the annotated gene ECs5115, and this overlapping gene pair is a novel example of this seemingly rare form of bacterial gene organization. We propose that *laoB* originated very recently by overprinting.

## Methods

Bacterial strains and plasmids used in this study are listed in Additional file 1. Oligonucleotides are listed in Additional file 2.

### Determination of transcriptional start site by 5′ rapid amplification of copy-DNA ends (RACE)

An overnight culture of *Escherichia coli* O157:H7 strain Sakai (GenBank accession NC_002695, EHEC) [22] was inoculated 1:100 in 0.5 × LB with 400 mM NaCl and incubated at 37 °C and 150 rpm until an OD_600_ of 0.8 was reached. Total RNA of 500 μl EHEC culture was isolated with Trizol and the remaining DNA was digested using 2 U TURBO™ DNase (Thermo Fisher Scientific). The 5’RACE System for Rapid Amplification of cDNA Ends, Version 2.0 (Invitrogen) was used according to the manual. After the second polymerase chain reaction (PCR), the dominant product was excised from the agarose gel and purified with the GenElute™ Gel Extraction Kit (Sigma-Aldrich). The PCR product was Sanger sequenced by Eurofins with oligonucleotide *laoB*+25R.

### Determination of transcriptional stop site by 3’RACE

Total RNA of 500 μl EHEC overnight culture in LB medium was isolated using Trizol and the remaining DNA was digested using 2 U TURBO™ DNase (Thermo Fisher Scientific). The 5′/3’ RACE Kit, 2nd Generation (Roche Applied Science) was applied according to the manual, but instead of an oligo-dT primer for cDNA synthesis the gene specific primer *laoB*-12F was used. A nested PCR was performed for product amplification. The dominant product was excised from the agarose gel, purified with the GenElute™ Gel Extraction Kit (Sigma-Aldrich) and Sanger sequenced (Eurofins) with oligonucleotide *laoB*+31F.

### Cloning of pProbe-NT plasmids and determination of promoter activity

The genomic region 300 bp upstream of the transcriptional start site was amplified by PCR and restriction enzyme cut sites for *Sal*I and *EcoR*I were introduced. The PCR products were cloned into the plasmid pProbe-NT [23] and transformed into *Escherichia coli* Top10. The plasmid sequence was verified by Sanger sequencing (Eurofins). Overnight cultures of *E. coli* Top10 + pProbe-NT (negative control) and pProbe-NT-PromoterTSS were used for 1:100 inoculation of 10 ml 0.5 × LB medium with 30 μg/ml kanamycin. The following conditions were investigated for promoter activity in 0.5 × LB medium each: plain LB, at pH 5, at pH 8.2, plus 400 mM NaCl, plus 0.5 mM CuCl_2_, plus 2 mM formic acid, plus 2.5 mM malonic acid, or plus 10 mM L-arginine. Cultures were incubated at 37 °C and 150 rpm until an OD_600_ of 0.5 was reached. Then, the cells were pelleted, washed once with phosphate-buffered saline (PBS) and resuspended in 1 ml PBS. The OD_600_ was adjusted to 0.3 and 0.6. Four-times each 200 μl of both OD-adjusted suspensions were pipetted in a black microtiter plate and the fluorescence was measured (Wallac Victor^3^, Perkin Elmer Life Science, excitation 485 nm, emission 535 nm, measuring time 1 s). The fluorescence of *E. coli* Top10 without vector was subtracted as background. To measure promoter activity after L-arginine supplementation, the experiment was repeated in depleted modified *Bacillus*-growth (MOD) medium [24] without L-glutamic acid, L-arginine, and L-aspartic acid, since these AA are easily convertible within the cell. Depleted MOD medium and depleted MOD medium supplemented with 10 mM L-arginine were tested. The experiments were performed in triplicate. Significance of changes was calculated by the two-tailed Student’s t-test.

### Cloning of a C-terminal LaoB-EGFP fusion protein and overexpression of LaoB-EGFP protein

The *laoB* sequence without the stop codon was amplified by PCR and restriction enzyme cut sites for *Pst*I and *Nco*I were introduced. The PCR product was cloned into the plasmid pEGFP and transformed into *Escherichia coli* Top10. The plasmid sequence was verified by Sanger sequencing (Eurofins). For overexpression of the fusion protein, overnight cultures of *E. coli* Top10 + pEGFP and *E. coli* Top10 + pEGFP-*laoB* were inoculated 1:100 in 10 ml 0.5 × LB medium with 120 μg/ml ampicillin in duplicates. Cultures were incubated at 37 °C and 150 rpm until an OD_600_ of 0.3 was reached. For one culture each, protein expression was induced using 10 mM isopropyl-β-D-1-thiogalactopyranoside (IPTG). Incubation of induced and uninduced cultures was continued for 1 h. Cells were pelleted, washed once with PBS and the pellet was resuspended in 1 ml PBS. The OD_600_ was adjusted to 0.3 and 0.6. Four times each 200 μl of the OD-adjusted bacterial suspensions were pipetted in a black microtiter plate and the fluorescence was measured as before. The experiment was performed in triplicate. Significance of changes was calculated by the two-tailed Student’s t-test.

### Cloning of a translationally arrested *laoB* mutant

For cloning of the genomic knock-out mutant ∆*laoB*, the method described by Kim et al. [25] was adapted. The mutations introduced do not change the AA sequence of the overlapping gene ECs5115. The pHA_1887_ fragment and the selection cassette were amplified by PCR from the plasmid pTS2Cb. Three consecutive point mutations, leading to a premature stop codon (5th codon) and a restriction enzyme cut site deletion (see below), were introduced into the *laoB* sequence by PCR using the oligonucleotides HA3*laoB*-139F and SM5*laoB*mut+42R (3′ mutation fragment) and SM3*laoB*mut-16F and HA5*laoB*+183R (5′ mutation fragment). Because the plasmid pTS2Cb-∆*laoB* was obtained by Gibson Assembly, the four PCR fragments contain overlapping sequences. In a total reaction volume of 20 μl, 200 fmol of each PCR fragment and the NEBuilder® HiFi DNA Assembly Master Mix (NEB) were incubated at 50 °C for 4 h. Two μl of the reaction were transformed into *E. coli* Top10 and plated on LB agar with 120 μg/ml ampicillin and 20 μg/ml chloramphenicol. Next, the mutation cassette was amplified by PCR using pTS2Cb-∆*laoB* as template and the PCR product of correct size was purified from an agarose gel (GenElute™ Gel Extraction Kit; Sigma-Aldrich). EHEC [22] was transformed with the plasmid pSLTS and, subsequently, transformed with 75 ng of the mutation cassette. After incubation for 3 h at 30 °C and 150 rpm in SOC medium, the cells were plated on LB-agar plates with 120 μg/ml ampicillin and 20 μg/ml chloramphenicol and incubated at 30 °C. One colony per plate was suspended in PBS. One-hundred μl of a 1:10 dilution in PBS were plated on LB agar with 120 μg/ml ampicillin and 100 ng/ml anhydrotetracycline for I-SceI induction and incubated at 30 °C over night. Several colonies were streaked on LB agar with 20 μg/ml chloramphenicol and plain LB agar and incubated at 37 °C over night. Colonies that were only able to grow on LB were selected and the genomic area surrounding the point mutations introduced was amplified by PCR. Additional to the premature stop codon, the restriction enzyme cut site for *Mnl*I was deleted, which was screened for by restriction digest of PCR products with this enzyme. Correct introduction of the three point mutations was assumed for *Mnl*I-digestion negative PCR products and confirmed by Sanger sequencing (Eurofins).

### Competitive growth assays

Overnight cultures in LB medium of EHEC wild type and EHEC ∆*laoB* were adjusted to an OD_600_ of 1.0 and then mixed in equal quantities (500 μl wild type + 500 μl mutant). Five-hundred μl of the mixture were pelleted and the cells were snap frozen in liquid nitrogen (control, t = 0). Ten ml 0.5 × LB medium were inoculated 1:3000 with the mixed EHEC culture. The following conditions were investigated in 0.5 × LB: plain LB, at pH 5, at pH 8.2, plus 400 mM NaCl, plus 0.5 mM CuCl_2_, plus 2 mM formic acid, plus 2.5 mM malonic acid, plus 4 mM malic acid, plus 400 μM ZnCl_2_, or plus 20 mM L-arginine. Cultures were incubated for 18 h at 37 °C and 150 rpm. Then, 500 μl of the culture were pelleted, 100 μl water were added to the pellet and the sample was heated to 95 °C for 10 min. Using this crude DNA preparation, a PCR was performed with the primer pair *laoB*-38F and *laoB*+140R. The PCR product was Sanger sequenced (Eurofins) and the ratio between wild type and mutant ∆*laoB* was determined by comparing peak heights. The absolute numbers were transformed into percentage values for each condition and the values were normalized to a t = 0 ratio for 50:50 wild type over mutant. Thus, the competitive index (CI) was calculated using the following formula:

$$ CI=\frac{mutant_{end}\left[\%\right]\times {wild\ type}_{start}\left[\%\right]}{mutant_{start}\left[\%\right]\times {wild\ type}_{end}\left[\%\right]} $$

The experiment was performed in biological triplicates. Significance was calculated by the two-tailed Student’s t-test.

### Complementation of EHEC ∆*laoB*

To compensate the *laoB* genomic knockout mutation, the intact *laoB* ORF was supplemented *in trans* on a plasmid. First, the sequence of *laoB* was amplified by PCR and restriction enzyme cut sites for *Nco*I and *Hind*III were introduced. The PCR product was cloned into the plasmid pBAD/*Myc*-*His*-C and the plasmid was transformed into EHEC ∆*laoB*. As a negative control, the plasmid containing the mutated *laoB* gene (∆*laoB*) was cloned. Next, competitive growth experiments were performed as described above using EHEC ∆*laoB* + pBAD-*laoB* (complementation) and EHEC ∆*laoB* + pBAD-∆*laoB* (control)*.* Both overnight cultures were supplemented with 120 μg/ml ampicillin and the cultures were mixed in equal ratio. Ten ml of either 0.5 × LB or 0.5 × LB + 20 mM L-arginine were inoculated 1:3000 in quadruplicates. Induction of the *laoB* frame (present either as wild type or as ∆*laoB*) was performed with 0.002% arabinose. After incubation at 37 °C and 150 rounds per minute (rpm) for 18 h, plasmids were isolated using the GenElute™Plasmid Miniprep Kit (Sigma-Aldrich). Using 20 ng isolated plasmid, PCR was performed with the oligonucleotides pBAD+208F and pBAD+502R. The PCR products were Sanger sequenced (Eurofins) and the ratio of intact *laoB* over translationally arrested ∆*laoB* was determined in percent. The experiment was performed in biological triplicates. Significant changes were calculated by the two-tailed Student’s t-test.

### Transcriptome and translatome sequencing

RNAseq and RIBOseq data sets of Hücker et al. [26] were investigated with respect to translated ORFs located in antisense to annotated genes. Briefly, the bacteria had been grown under the following growth conditions: LB medium at 37 °C, harvested at OD_600_ 0.4, BHI medium at 37 °C, harvested at OD_600_ 0.1, and BHI medium supplemented with 4% NaCl at 14 °C, harvested at OD_600_ 0.1. An ORF was considered translated, when (i) it was covered with at least one read per million mapped sequenced reads normalized to 1 kbp, (ii) ≥ 50% of the ORF is covered with RIBOseq reads, and (iii) the ribosomal coverage value (RCV) is at least 0.25 in both biological replicates. All three requirements were matching for *laoB*, which was verified by visual inspection using the Artemis genome browser [27].

### Bioinformatics methods to characterize *laoB*

#### Prediction of σ^70^ promoters

The region 550 bp upstream of the start codon of *laoB* was searched for the presence of a σ^70^ promoter with the program BPROM (Softberry) [28]. The linear discriminant functions (LDF) score given is a measure of promoter strength, whereupon an LDF score of 0.2 indicates presence of a σ^70^ promoter with 80% accuracy and specificity.

#### Prediction of alternative σ-factors

The search for alternative σ-factors was performed manually. The sequence 50 bp upstream of the detected transcription start site (TSS) was compared to the consensus motifs of σ^28^ [29], σ^32^ [30], and σ^54^ [31].

#### Prediction of ρ-independent terminators

The region 300 bp downstream of the stop codon of *laoB* was searched for the presence and folding energy of a ρ-independent terminator using the program FindTerm (Softberry) [28].

#### Prediction of Shine-Dalgarno (SD) sequence

The free energy ∆G° of the region 30 bp upstream of the start codon of *laoB* was calculated according to Ma et al. [32]. The perfect SD sequence taAGGAGGt has a ∆G° of − 9.6. A ∆G° of − 2.9 is considered the threshold for the presence of an SD sequence [32].

#### Detection of annotated homologs

The AA sequence LaoB, corresponding to *laoB*, was used to query the data base GenBank with blastp using default parameters [33].

#### PredictProtein

LaoB was submitted to the software PredictProtein [34]. The methods PROFphd (secondary structure) [35], TMSEG (transmembrane helices) [36], DISULFIND (disulfide bonds) [37] and LocTree2 (subcellular localization) [38] were used.

#### Phylogenetic tree construction

For evolutionary analysis of *laoB* and ECs5115, tblastn was used with an e-value cutoff of 0.001 and at least 50% identity, which allows a search of nucleotide sequences homologous to a protein sequence query in all genomic sequences of the database independent of their annotation status [39, 40]. For the short gene of LaoB, tblastn was not sensitive enough to detect all existing genomic sequences; hence, hits matching ECs5115 were used for subsequent *laoB* analysis. Continuous *laoB* ORFs were detected in a total of 497 *Escherichia* and 18 *Shigella* strains (see results). However, a large number of genes had the very same *laoB* sequence. Thus, examples of 11 LaoB-encoding sequences, representing the diversity of continuous *laoB* genes, were chosen. Likewise, exemplary ECs5115 sequences within a broad range of different sequence identities were downloaded from the database and used for phylogenetic analysis. Multiple sequence alignments of ECs5115 and LaoB homologs were conducted using MUSCLE implemented in MEGA6 [41]. The automated alignments were manually checked and adapted, where necessary. Parts encoding sequences homologs to LaoB were manually identified in −2 frame of ECs5115. Those sequences with no obvious similarity to *laoB* were identified by pairwise alignments of the nucleotide sequence of the −2 frame of the respective ECs5115 homolog (EMBOSS Needle, [42]). The area which aligned to *laoB* was translated to its AA sequence and further aligned outside the initial region by multiple sequence alignments, as before. The *laoB* sequence was only found to be discontinuous outside the *Escherichia/Shigella* clade. Thus, indel-like sequence insertions and internal stop codons are present in sequences of bacteria outside *Escherichia/Shigella,* encoding peptide fragments shorter than 41 AA or AA sequences which are very different from LaoB.

Reference phylogenetic trees of the strains and species examined were constructed according to Fellner et al. [14]. Briefly, a concatenated sequence of the housekeeping genes 16S rDNA*, atpD, adk, gyrB, purA* and *recA* was used. The sequences were aligned using ClustalW in MEGA6. Columns with gaps or ambiguities were removed. The final dataset contains 7484 positions. The best nucleotide substitution model was searched for using MEGA6. The final Maximum-Likelihood tree was calculated using Neighbor Joining and bootstrapped 1000 times. The best nucleotide substitution model for tree construction was identified to be the General Time reversible model (GTR with a lowest Bayesian Information Criterion of 123,336.358). The non-uniformity of evolutionary rates among substitution sites was modeled using a discrete Gamma distribution with five rate categories (+G, parameter = 0.5494). The log likelihood value of the final tree was − 61,963.20.

## Results

### Detection of a transcribed and translated antiparallel overlapping ORF

**Fig. 1:**
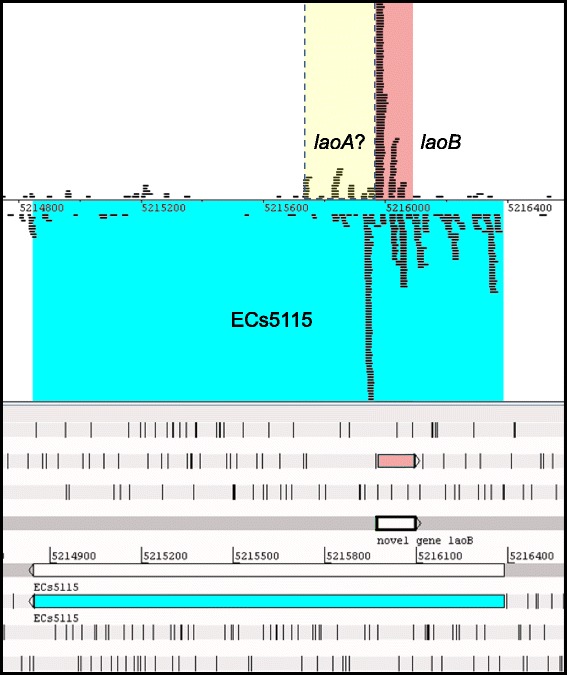
Translation of *laoB* in LB medium*.* RIBOseq reads mapped strand-specifically to the overlapping gene pair *laoB*/ECs5115 are visualized in Artemis. The annotated gene ECs5115 is highlighted in blue. The novel gene *laoB* is highlighted in pink. A potential, non-characterized overlapping gene *laoA* is highlighted in yellow

Combined RNAseq and RIBOseq data of EHEC grown at three different growth conditions were searched for ORFs, which are antiparallel overlapping to annotated genes and show signals for transcription and translation. Further, the translatability of the ORFs was calculated using the RCV, which is defined as the quotient of reads per kilobase per million mapped reads (RPKM) of the translatome over transcriptome RPKM [43]. The annotated ECs5115 (1539 bp) encodes a transcriptional regulator of the CadC family and shows signals for transcription (RNAseq) and translation (RIBOseq) on the sense and antisense strand (Fig. 1). The latter reads correspond to a small ORF completely embedded within ECs5115 in the reading frame −2 relative to ECs5115, encoding a short hypothetical protein of 41 AA. A blastp search for annotated homologs resulted in a single hit to a hypothetical protein of *Escherichia albertii* TW07627 (GenBank accession EDS93387.1) with an e-value of 5 × 10^− 13^ and 78% identity. The software PredictProtein could not detect transmembrane helices or disulfide bonds and the hypothetical protein is predicted to be secreted. The strongest transcription of the ORF was found in BHI medium at 37 °C (Table 1A), whereas translation (RPKM) and translatability (RCV) are highest in LB at 37 °C. ECs5115 is only weakly transcribed and read numbers decrease over the length of the gene (Fig. 1 and Table 1B). Translation of both reading frames, ECs5115 and *laoB* is almost completely switched off at combined cold and osmotic stress.

**Table 1:** Transcription and translation of *laoB* (part 1A) and its mother gene ECs5115 (part 1B) at the three different growth conditions indicated. The RPKM values of the transcriptome (RNAseq) and the translatome (RIBOseq) data for the overlapping novel gene and annotated mother gene are listed, including the RCV, indicating their translatability. ORF coverage is the fraction of a gene sequence, which is covered by RIBOseq reads. In addition, the corresponding data for the putative overlapping gene *laoA* (compare Fig. 2a) are shown (part 1C)

Condition	RPKM transcriptome^a^	RPKM translatome^a^	RCV^a^	ORF coverage^a^
**1A** *laoB*				
LB, 37 °C	29.5	194	6.83	0.7
BHI, 37 °C	49.4	23.6	0.48	0.6
BHI + 4% NaCl, 14 °C	28.3	0.2	0.01	0.07
**1B** ECs5115				
LB, 37 °C	19	12.3	0.65	0.35
BHI, 37 °C	26.9	7.1	0.27	0.45
BHI + 4% NaCl, 14 °C	10.2	0.6	0.07	0.16
**1C** *laoA*				
LB, 37 °C	38.3	9.6	0.27	0.51
BHI, 37 °C	36.3	1.9	0.05	0.37
BHI + 4% NaCl, 14 °C	12.7	0.4	0.02	0.16

^a^Mean values of the two biological replicates are shown

### Characterization of *laoB* promoter region

**Fig. 2:**
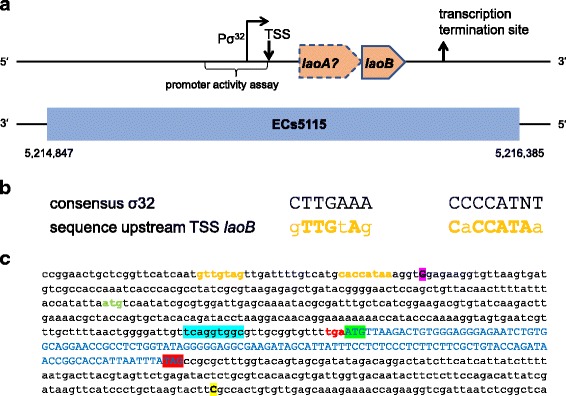
The overlapping gene pair *laoB*/ECs5115 and the regions upstream and downstream. **a** Schematic view of the overlapping gene pair *laoAB*/ECs5115. Positions of the promoter, TSS, and transcription termination are indicated (not to scale). **b** Alignment of the proposed σ^32^ promoter to the consensus motif. **c** DNA sequence of the novel gene *laoB* and the upstream and downstream regions. The sequence of *laoB* is colored in blue and written in capital letters. The start codon is highlighted in green and the stop codon in red. Also the start and stop codon of the potential upstream gene *laoA* are marked (lower case, green and red, respectively). The TSS detected by 5’RACE is highlighted in pink and the transcriptional stop determined by 3’RACE is highlighted in yellow. The putative σ^32^ promoter is colored in orange. The SD sequence upstream *laoB* is highlighted in light blue

A predicted SD sequence (∆G° of − 6.8) is present 15 bp upstream of the putative start codon (Fig. 2c). A single transcriptional start site was identified 289 bp upstream of the start codon by 5’RACE. This would imply a very long 5′ untranslated region (UTR). Therefore, the region was searched for additional ORFs. Indeed, another ORF (*laoA*), which would encode a protein of 61 AA, is located directly upstream of the OLG *laoB* (Fig. 2a). However, this ORF is only weakly translated (Fig. 1 and Table 1C). Furthermore, it does not have annotated homologs, and in its upstream region, no SD sequence was detected. Thus, this ORF was not characterized further. While FindTerm does not predict a ρ-independent terminator in the region 300 bp downstream of the stop codon, a transcriptional stop site was determined 155 bp downstream of the stop codon by 3’RACE (Fig. 2c).

**Fig. 3:**
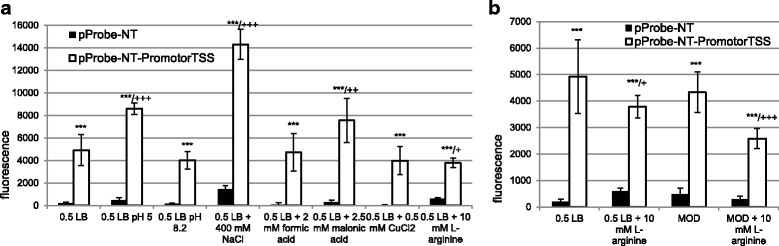
Promoter activity of the region 300 bp upstream of the *laoB* TSS. Significant changes between vector control and pProbe-NT-PromoterTSS are marked with asterisks (*** *p* < 0.001). Significant differences between 0.5 × LB and investigated stress conditions are marked with a plus (+ *p* < 0.05, ++ *p* < 0.01, +++ *p* < 0.001). **a** Promoter activity of the region 300 bp upstream of the determined TSS in LB medium with different supplementations. **b** Promoter activity of the region 300 bp upstream of the TSS in LB or depleted MOD medium and LB or in depleted MOD medium supplemented with 10 mM L-arginine

The software BPROM did not predict a σ^70^ promoter in the upstream region of *laoB* in a suitable distance to the TSS. Therefore, the region upstream of the TSS was manually investigated for the presence of alternative σ-factor consensus motifs. Interestingly, a sequence with high similarity to the σ^32^ consensus motif was detected in proper distance to the TSS (Fig. 2b). The sequence 300 bp upstream of the TSS, containing the potential σ^32^ promoter, was cloned into pProbe-NT for investigation of promoter activity at different growth conditions. Significant promoter activity was detectable at all conditions tested (Fig. 3a). LB supplemented with 400 mM NaCl lead to the highest fluorescence intensity with a 2.9-fold increase compared to LB. Additionally, the conditions LB + 2.5 mM malonic acid and LB at pH 5 showed a significantly increased promoter activity. Promoter activity was reduced in LB supplemented with 10 mM L-arginine of about 1.3-fold. However, LB medium already contains L-arginine. Therefore, the experiment was repeated in depleted MOD medium without L-arginine and the convertible AA, which leads to a more pronounced decline of promoter activity (Fig. 3b).

### Expression of a LaoB-EGFP fusion protein

**Fig. 4:**
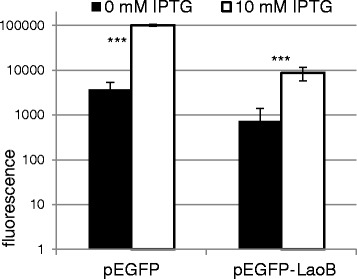
Overexpression of LaoB C-terminally fused to EGFP. *E. coli* Top10 was transformed with the empty pEGFP vector as positive control (left) and with pEGFP-LaoB (right). Fluorescence values in logarithmic scale without (black) and with (white) induction are depicted. Expression of the fusion protein was induced with 10 mM IPTG after adjusting the OD_600_ to 0.6. The experiment was performed in triplicate (*** *p* < 0.001)

Next, it was investigated whether the LaoB protein can be expressed in *E. coli.* For this purpose, the *laoB* sequence was cloned in-frame and upstream of EGFP, and transformed into *E. coli* Top10. After induction with IPTG, a fluorescent LaoB-EGFP fusion protein was produced. The induced culture shows an 11.7-fold increased fluorescence intensity compared to the uninduced culture demonstrating expression of the fusion protein (Fig. 4).

### The translationally arrested mutant ∆*laoB* shows a growth advantage in arginine-containing media

**Fig. 5:**
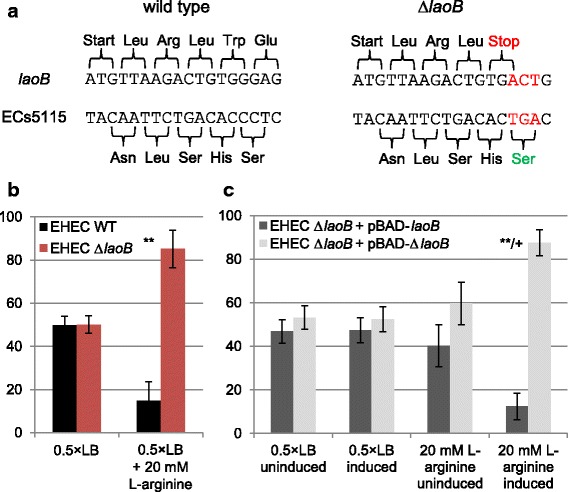
Nucleotide sequence and phenotype of EHEC ∆*laoB*. **a** Construction of a translationally arrested ∆*laoB* mutant. Introduction of a point mutation in the DNA sequence of *laoB* changed the fifth codon encoding glutamine to a premature stop codon. Because of two adjacent mutations, a cut site for the restriction enzyme *MnI*I is deleted at this position. The three point mutations do not influence the AA sequence of the antiparallel overlapping annotated gene ECs5115*.* **b** Ratio in percent of EHEC wild type to EHEC ∆*laoB* after competitive growth. Wild type and mutant were mixed in equal ratios and after 18 h incubation at different growth conditions, their ratio was determined. In 0.5 × LB, no change compared to the inoculation ratio occurred, but when the medium was supplemented with 20 mM L-arginine, EHEC ∆*laoB* shows a significant growth advantage. The experiment was performed in triplicate (** *p* < 0.01). **c** Complementation of EHEC ∆*laoB* using a plasmid-borne *laoB*. The diagram shows the ratios in percent of EHEC ∆*laoB* + pBAD-*laoB* and EHEC ∆*laoB* + pBAD-∆*laoB* after competitive growth*.* The experiment was performed in triplicate. Significant changes between uninduced and induced conditions are marked with a plus (+ *p* < 0.05). Significant changes between 0.5 × LB and 0.5 × LB + 20 mM L-arginine are marked with asterisks (** *p* < 0.01)

For functional characterization of *laoB* the knock-out mutant ∆*laoB* was created using genome editing [25]. A premature stop codon at the fifth codon of *laoB* was generated by a point mutation (Fig. 5a). Two additional point mutations were introduced in adjacent nucleotides to delete an *MnI*I restriction enzyme cut site (required for easier selection). The AA sequence of ECs5115 is not changed by the point mutations, because the affected codon of the mother gene still encodes serine.

To find a potential phenotype, competitive growth experiments with EHEC wild type and ∆*laoB* were performed. The equal-ratio mixture of wild type and mutant was incubated under different conditions and a potential growth advantage was determined by the ratio of the wild type and mutant genes at the endpoint. When LB medium was supplemented with 20 mM L-arginine, a phenotype was detected: EHEC ∆*laoB* displayed a significant growth advantage indicated by a ratio of wild type to mutant of 15:85 (Fig. 5b). Thus, the CI is 13.6. No phenotype was found for any other conditions tested (Additional file 3).

Intact *laoB*, cloned into pBAD-*myc-His*-C, should restore the phenotype of EHEC wild type. Therefore, competitive growth experiments using EHEC ∆*laoB* carrying pBAD-*laoB* against EHEC ∆*laoB* + pBAD-∆*laoB* (mutant control) were performed. Expression of *laoB* was induced with arabinose. As expected, in plain LB, the ratio between the two strains tested did not change independent whether the plasmid borne *laoB* was induced or not (Fig. 5c). In contrast, in LB supplemented with 20 mM L-arginine, the strain carrying the functional *laoB*-copy shows a significant growth disadvantage if induced with arabinose. This reflects the competitive growth phenotype of the wild type strain compared to the mutant strain (Fig. 5b). Thus, translation arrested *laoB* can be complemented *in trans.*

### Phylostratigraphic analysis of *laoB*

**Fig. 6:**
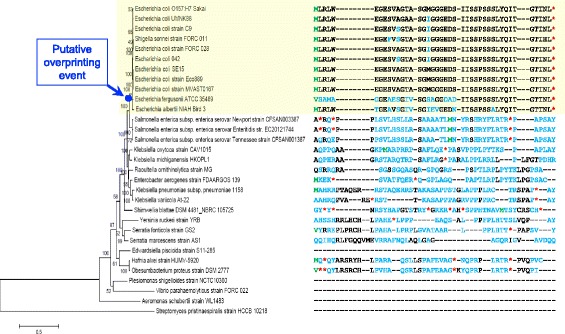
Phylogenetic tree and alignment of *laoB*. The phylogenetic tree to the left was constructed based on a concatemer of 16S rDNA, *atpD*, *adk*, *gyrB*, *purA*, and *recA.* To the right, the different AA sequences of LaoB are aligned. Start codons are colored in green, AA changes in blue, and stop codons (*) in red

Two tblastn searches with ECs5115 and *laoB* as queries were performed to determine the taxonomic distribution of both genes. A continuous *laoB*-encoding gene was detected only in *Escherichia* and *Shigella* strains (Fig. 6 and Additional file 4). The antiparallel overlapping, annotated ECs5115 has further homologs in many bacterial phyla outside *Escherichia/Shigella* (Additional file 5). However, in those sequences, the embedded *laoB* ORF is either quite different in the encoded AA sequence compared to the original *laoB* or composed of uncontinuous segments. Thus, either stop codons break the *laoB* frame or frame-shift-like modifications are causing observed differences. When a continuous *laoB* homolog is present, its sequence is always highly conserved, showing only a few AA substitutions, but neither premature stop codons nor frameshift mutations. The highest sequence variability in intact LaoB homologs occurs in *E. fergusonii* (Fig. 6).

## Discussion

This study provides evidence for a novel overlapping gene pair, *laoB*/ECs5115, in EHEC. Transcription and translation of a short ORF, embedded in the antisense reading frame −2 to a CadC-like transcriptional regulator, was detected by RNAseq and RIBOseq at optimal growth conditions. Translational knockout of the ORF by a premature stop codon resulted in a significant growth advantage of the mutant strain in LB medium supplemented with L-arginine over the wild type strain in competitive growth. Consistently, the activity of the putative σ^32^ promoter is repressed by L-arginine. Whether *laoB* is part of an overlapping operon together with *laoA*, located upstream of *laoB*, is unknown. *LaoA* was not examined, since transcription and translation of *laoA* appeared to be very weak under the conditions tested*.*

### Is *laoB* a protein-coding gene?

*LaoB* might function as a novel non-coding RNA (ncRNA) instead of a novel protein-coding gene. However, due to the following reasons this appears to be unlikely: Most important, experimental data presented here confirm the protein-coding character of *laoB*, since the ORF is covered by RIBOseq reads (Fig. 1). RIBOseq signals clearly indicate active translation of an RNA molecule [44, 45]. In LB medium, the ORF has a very high RCV (Table 1A), which is much higher than the mean RCV of 1.55, which we found for short annotated EHEC genes [26, 43]. In addition, stable translation into a protein was further confirmed by the expression of a LaoB-EGFP fusion protein (Fig. 4). Second, a translationally arrested mutant lead to a clear phenotype which could be complemented by the wild type sequence *in trans* by using just the *laoB* ORF without any adjacent sequence attached (Fig. 5). If *laoB* would function as an antisense ncRNA, it would regulate its targets by base pairing with complementary mRNAs [46]. It appears to be unlikely that a translationally arrested mutant, which changes only ~ 0.5% of the nucleotides compared to the complete transcript of the *laoB* sequence, would exert such a dramatic phenotype. Third, 15 bp upstream of the start codon an SD sequence is present (Fig. 2c). The distance of the SD to the start codon is within the natural ranges observed and the detected sequence is close to the SD consensus motif, resulting in strong ribosomal binding [32]. Finally, the *laoB* ORF has been annotated in *E. albertii* as a protein-coding gene.

### Putative function of LaoB

The results presented in this work provide first hints towards a potential LaoB function. The region 300 bp upstream of the TSS determined shows significant promoter activity at all investigated conditions (Fig. 3). The *laoB* promoter is probably recognized by the alternative σ-factor σ^32^, since a sequence very similar to the σ^32^ consensus motif is present in the proper distance to the TSS (Fig. 2b) [30]. The first T of the −35 box and the A of the −10 box are completely conserved in σ^32^ promoters and both nucleotides are present in the σ^32^ promoter region of *laoB*. Additionally, the spacer between the −35 and −10 box has the optimal distance of 14 bp and σ^32^ promoters with this spacer distance tolerate a substitution of the second C of the tetra-C motif of the −10 box without losing promoter strength [47], which is also the case here. In addition, the distance between the −10 box and the TSS is in the optimal range of 6 bp [30]. Transcription of heat shock genes is induced by σ^32^. Accordingly, transcription of *laoB* is almost switched off at cold stress (Table 1A). The σ^32^ stress regulon includes chaperons, transcription factors, DNA/RNA surveillance proteins, and many membrane-associated proteins [30]. In this study, the promoter has the highest activity in LB supplemented with NaCl and at acidic conditions (Fig. 3). Interestingly, σ^32^ is also the master regulator of the transcription factor PhoPQ, which is also induced at acid stress [30].

In our hands, EHEC ∆*laoB* only showed a clear phenotype after supplementing the medium with L-arginine (Fig. 5b). As a proteinogenic AA, L-arginine is involved in many central metabolic pathways. Bacteria synthesize L-arginine from glutamate [48] or take it up from the environment by three different transporters [49]. Arginine can be utilized as sole carbon and nitrogen source and is the substrate for the synthesis of polyamines [48]. Here, high L-arginine concentrations resulted in a significantly reduced activity of the *laoB* promoter and the EHEC wild type has a clear growth disadvantage in competitive growth. These observations would agree with the speculation that LaoB might be involved in enhancing L-arginine uptake. In many EHEC reservoirs, nutrient concentrations, including L-arginine concentrations, are low and efficient uptake represents an advantage. The high arginine concentrations used in this study are unlikely to occur naturally. Therefore, under environmental conditions, which are low in arginine, intact LaoB may confer a growth advantage. The hypothesis that LaoB somehow interacts with arginine transport is supported by the facts that a high proportion of small proteins – LaoB has a size of only 41 AA - associates with the cell membrane, in which transporters are located [50, 51], and that the σ^32^ regulon includes many membrane proteins [30]. However, testing this speculation and further functional characterization of LaoB must await future studies.

### Origin of *laoB* by overprinting

The time of origin of an OLG can be estimated by phylostratigraphic analysis, comparing the phylogenetic distribution of the mother gene and the overlapping gene [18, 52]. The intact *laoB* ORF is only present in *Escherichia* and *Shigella* strains (Fig. 6) while the annotated gene ECs5115 has a much broader taxonomical distribution (i.e., higher conservation level) and is present in both Gram-negative and Gram-positive bacteria (Additional file 5). It is concluded that *laoB* originated recently and might be an interesting example of de novo gene birth by overprinting [18, 52, 53]. This would mean that a number of point mutations in the ECs5115 sequence would have created the *laoB* ORF including its regulatory sequences after the *Escherichia/Shigella* clade separated from *Salmonella*. One may postulate that a weak σ^32^ promoter sequence was already present at the proper location by chance and, later, may have been further optimized by additional point mutations leading to an increased transcription of the novel ORF. The resulting (m)RNA must have been used as template for translation, perhaps based on a weak ribosomal binding site which happened to be present upstream of the start codon. Now, one must assume that the AA chain, at this point, was functional ab initio by chance*,* conferring a fitness advantage to the cell. At this early evolutionary stage, a novel gene is volatile and the process is reversible, such that the novel ORF can get lost again [54]. A fitness gain related to the L-arginine metabolism may have led to fixation of the functional allele in the population by Darwinian evolution. Because EHEC colonizes many hosts and environments [55], which requires expression of different sets of genes [56, 57], LaoB might improve its fitness in one of those species specific niches. Alternatively, the novel ORF could have been fixed by neutral evolution together with the mutated mother gene [58]. Later on, extension at the 3′ end by the loss of a stop codon may occur, leading to an elongation of the novel protein which would be more likely than 5′ elongation due to regulatory elements in the 5’UTR [59]. This speculative order of events has some similarities to the proto-gene hypothesis of Carvunis et al. [39], which deals with the potential de novo origin of short genes in intergenic regions of the yeast *S. cerevisiae*.

In EHEC, only two other antiparallel overlapping gene pairs, in which a young gene also may have originated recently de novo by overprinting, have been characterized functionally [13, 53]. For *E. coli* K12 two additional antiparallel overlapping gene pairs are described, *yghX/modA* [60] and *tnpA/astA* [61] respectively, which might have also originated by overprinting. Another OLG pair exists in *Streptomyces coelicolor*: The knock-outs of the antiparallel overlapping genes *dmdRI* and *adm* both show a phenotype [11]. In addition, in *Bacillus subtilis* an annotated OLG pair exists [62].

Whether de novo birth of genes in antisense to annotated genes is more frequent than presumed is still open for discussion, but has been suggested by Haycocks and Grainger [63] based on the frequent binding of transcriptional regulators in intragenic locations. In contrast, a gene duplication followed by neofunctionalization or subfunctionalization, which is the established theory for the origin of new genes [20], produces just variants of existing sequences, overprinting would allow for the rapid creation of true novelty [64].

## Conclusion

Strand-specific RNAseq and RIBOseq are well suited to identify translated ORFs located in antisense to annotated genes. Frequent antisense transcription is observed in all RNAseq experiments, but almost all signals have been interpreted as ncRNA [65]. However, RIBOseq already confirmed translation of many antisense RNAs in eukaryotes [66–68], and this method identified numerous overlooked small genes in the intergenic regions of different bacteria [69–71]. Therefore, improved genome annotation algorithms are required which do not systematically dismiss small and/or overlapping genes [8, 72, 73]. Integration of transcriptomic, translatomic, and other experimental data into annotation pipelines would increase specificity and sensitivity for the prediction of novel small genes [74–76]. Additionally, improved proteomic methods are necessary, which do not miss small non-annotated proteins [77, 78]. In any case, functional characterization of novel short genes overlooked to date presents a major future challenge to experimental microbiology. In this paper, we provide initial functional characterization and evidence for overprinting of a small protein encoded in antisense to an annotated protein-coding gene. We assume such overprinting events could be significant for EHEC (e.g., [13, 14]) and maybe other bacteria.

## Additional files

Additional file 1: Table S1. Bacterial strains and plasmids used in this study. (DOCX 16 kb)

Additional file 2: Table S2. Oligonucleotides used in this study. Restriction enzyme cut sites are highlighted in bold. (DOCX 19 kb)

Additional file 3: Ratio in percent of EHEC wild type to EHEC ∆*laoB* after competitive growth at different growth conditions. Neither the wild type nor the mutant show a significant growth advantage at any of the depicted conditions. The experiment was performed in triplicate. (PPTX 44 kb)

Additional file 4: Phylogenetic analysis of *laoB* at the DNA level. Start codons are colored in blue and stop codons in purple. (PPTX 1172 kb)

Additional file 5: Phylogenetic analysis of ECs5115 by the Maximum Likelihood method. The tree with the highest log likelihood (− 61,042.2643) is shown. The percentage of trees in which the associated taxa clustered together is shown next to the branches. Initial tree(s) for the heuristic search were obtained by applying the Neighbor-Joining method to a matrix of pairwise distances estimated using the Maximum Composite Likelihood (MCL) approach. A discrete Gamma distribution was used to model evolutionary rate differences among sites (5 categories; +*G*, parameter = 0.5123). The rate variation model allowed some sites to be evolutionarily invariable (+*I*, 32.8153% sites). The tree is drawn to scale, with branch lengths measured in the number of substitutions per site. The analysis involved 30 nucleotide sequences. All positions containing gaps and missing data were eliminated. There was a total of 8025 positions in the final dataset. Evolutionary analyses were conducted in MEGA6. In the alignment to the right, start codons are highlighted in blue and stop codons with a red asterisk. (PPTX 614 kb)

## Abbreviations

AA: Amino acid(s)
BHI: Brain-heart infusion
bp: Base pair(s)
CI: Competitive index
EGFP: Enhanced green fluorescent protein
IPTG: Isopropyl-β-D-1-thiogalactopyranoside
*laoB*: L-arginine responsive overlapping gene
LB: Luria-Bertani
LDF: Linear discriminant functions
MOD medium: Modified *Bacillus*-growth medium
ncRNA: non-coding RNA
OD_600_: Optical density at 600 nm
OLG: Overlapping gene
ORF: Open reading frame
PBS: Phosphate-buffered saline
PCR: Polymerase chain reaction
RACE: Rapid amplification of copy-DNA ends
RCV: Ribosomal coverage value
RIBOseq: Ribosomal-footprint sequencing
RNAseq: RNA sequencing
RPKM: Reads per kilobase per million mapped reads
rpm: Rounds per minute
SD: Shine-Dalgarno
TSS: Transcription start site
UTR: Untranslated region
