# Supplementary material for: A novel short L-arginine responsive protein-coding gene (laoB) antiparallel overlapping to a CadC-like transcriptional regulator in Escherichia coli O157:H7 Sakai originated by overprinting
Source: BMC Evol Biol. 2018 Feb 12;18:21. doi: 10.1186/s12862-018-1134-0 (PMC5810103; doi:10.1186/s12862-018-1134-0)
Supplement: Supplementary file 1 — Bacterial strains and plasmids used in this study. (DOCX 16 kb) [file 12862_2018_1134_MOESM1_ESM.docx]

| strain or plasmid | characteristics | reference |
| --- | --- | --- |
| *Escherichia coli* O157:H7 Sakai (EHEC) | wildtype, outbreak strain | Hayashi et al., 2001 |
| *Escherichia coli* O157:H7 Sakai ∆*laoB* | translational arrested mutation of *laoB* | this study |
| *Escherichia coli* Top10 | F-, mcrA, Δ(mrr-hsdRMS-mcrBC), φ80lacZΔM15, ΔlacX74, nupG, recA1, araD139, Δ(ara-leu)7697, galE15, galK16, rpsL(Str^R^), endA1, λ^-^ | Invitrogen |
| pProbe-NT | pBBR1 replicon, *gfp* reporter, Km^R^ | Miller et al., 2000 |
| pProbe-NT-PromotorTSS | 300 bp upstream of *laoB* TSS | this study |
| pEGFP | pUC ori, P_lac_, *egfp* reporter, Amp^R^ | CLONTECH Laboratories |
| pEGFP-*laoB* | C-terminal EGFP-fusion protein | this study |
| pSLTS | derivate of pKDTS | Kim et al., 2014 |
| pTS2Cb | derivate of pUC19, I-SceI, Amp^R^, Cm^R^ | Kim et al., 2014 |
| pTS2Cb-∆*laoB* | premature stop codon in *laoB* | this study |
| pBAD/*Myc*-*His*-C | derivate of pBR322, *araBAD* promoter, *myc C*-tag and his-tag fusion, Amp^R^ | Invitrogen |
| pBAD-*laoB* | complementation | this study |
| pBAD-∆*laoB* | complementation | this study |

**Supplementary Table S1:** Bacterial strains and plasmids used in this study.
