# Supplementary material for: A novel short L-arginine responsive protein-coding gene (laoB) antiparallel overlapping to a CadC-like transcriptional regulator in Escherichia coli O157:H7 Sakai originated by overprinting
Source: BMC Evol Biol. 2018 Feb 12;18:21. doi: 10.1186/s12862-018-1134-0 (PMC5810103; doi:10.1186/s12862-018-1134-0)
Supplement: Supplementary file 2 — Oligonucleotides used in this study. Restriction enzyme cut sites are highlighted in bold. (DOCX 19 kb) [file 12862_2018_1134_MOESM2_ESM.docx]

| name | sequence 5’- 3’ | purpose |
| --- | --- | --- |
| *laoB*-570F-*Sal*I | tagt**GTCGAC**cgcaccacgatgaggcaaaat | promotor activity upstream TSS |
| *laoB*-291R-*EcoR*I | cgtg**GAATTC**acctttatggtgcatgacaa | promotor activity upstream TSS |
| *laoB*+95R | tgccggttatctggtaca | 5’RACE, reverse transcription |
| *laoB*+59R | gggagaggaaataatgctatcttcgc | 5’RACE, 1^st^ PCR |
| *laoB*+25R | cagaggcggttcctgccacaga | 5’RACE, 2^nd^ PCR |
| *laoB*-12F | ggattgttcaggtggcgttg | 3’RACE, reverse transcription |
| *laoB*+3F | gttaagactgtgggagggagaatct | 3’RACE, 1^st^ PCR |
| *laoB*+31F | gcaggaaccgcctctggtat | 3’RACE, 2^nd^ PCR |
| *laoB*+1F-*Pst*I | atct**CTGCAG**gatgttaagactgtgggaggg | EGFP-fusion protein |
| *laoB*+104R-*Nco*I | cgct**CCATGG**ctaaattaatggtgccggtta | EGFP-fusion protein |
| pHA5F | cgcaggaaagaacatgtg | amplification pHA1887 |
| pHA3R | aagggcctcgtgatacg | amplification pHA1887 |
| HA3F | aggcgtatcacgaggccctt | amplification mutation cassette |
| HA5R | ctcacatgttctttcctgcg | amplification mutation cassette |
| SM5F | atctcaagagtggcagcggt | amplification selection cassette |
| SM3R | ttatccacctccttgc | amplification selection cassette |
| HA3*laoB*-139F | aggcgtatcacgaggcccttagacgtgtatcaa gactt | translationally arrested ∆*laoB* mutant |
| SM5*laoB*mut+42R | accgctgccactcttgagatggcggttcctgcca cagattctcccagtcacag | translationally arrested ∆*laoB* mutant |
| SM3*laoB*mut-16F | gcaaggaggtgcataagttgcggtgttttgaatgtt aagactgtgactggga | translationally arrested ∆*laoB* mutant |
| HA5*laoB*+183R | ctcacatgttctttcctgcgagatagtcctgtctatat | translationally arrested ∆*laoB* mutant |
| *laoB*-38F | aactggggattgttcaggtg | amplification *laoB* |
| *laoB*+140R | gtaccaaagcgcggctataa | amplification *laoB* |
| *laoB*+1F-*Nco*I | tgca**ccatgg**gaatgttaagactgtgggaggg | complementation wildtype |
| *laoB*mut+1F-*Nco*I | actt**ccatgg**gaatgttaagactgtgactggg | complementation mutant |
| *laoB*+107R-*Hind*III | gctc**aagctt**ctataaattaatggtgccgg | complementation |
| pBAD+208F | atgccatagcatttttatcc | amplification pBAD-*myc-His*-C |
| pBAD+502R | ctgatttaatctgtatcagg | amplification pBAD-*myc-His*-C |

**Supplementary Table S2:** Oligonucleotides used in this study. Restriction enzyme cut sites are highlighted in bold.
