# Supplementary material for: A novel short L-arginine responsive protein-coding gene (laoB) antiparallel overlapping to a CadC-like transcriptional regulator in Escherichia coli O157:H7 Sakai originated by overprinting
Source: BMC Evol Biol. 2018 Feb 12;18:21. doi: 10.1186/s12862-018-1134-0 (PMC5810103; doi:10.1186/s12862-018-1134-0)
Supplement: Supplementary file 3 — Ratio in percent of EHEC wild type to EHEC ∆laoB after competitive growth at different growth conditions. Neither the wild type nor the mutant show a significant growth advantage at any of the depicted conditions. The experiment was performed in triplicate. (PPTX 44 kb) [file 12862_2018_1134_MOESM3_ESM.pptx]

## Slide 1
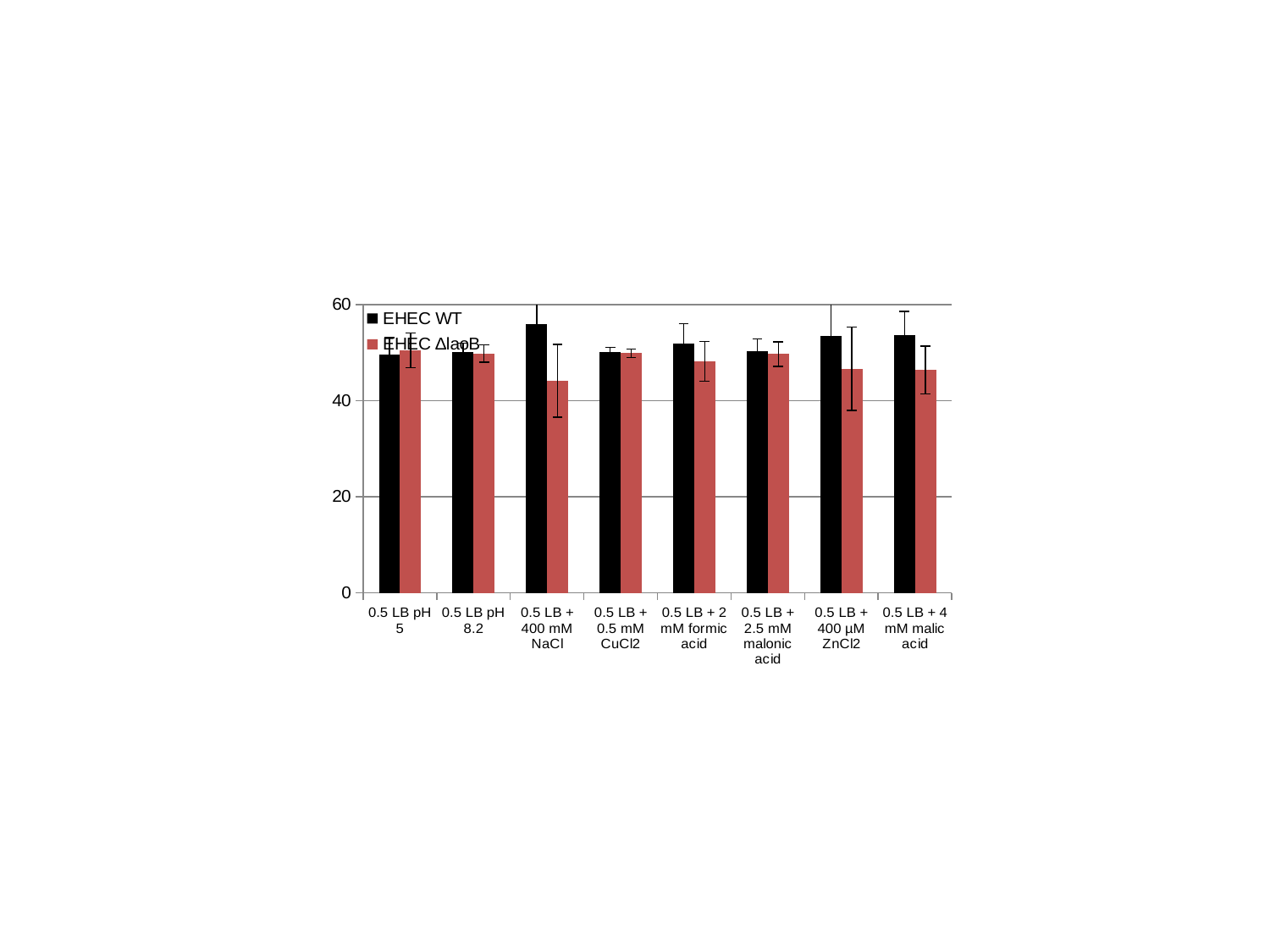

### Chart
| Category | EHEC WT | EHEC ∆laoB |
|---|---|---|
| 0.5 LB pH 5 | 49.51696634732082 | 50.48303365267918 |
| 0.5 LB pH 8.2 | 50.184600342721694 | 49.81539965727831 |
| 0.5 LB + 400 mM NaCl | 55.88648222859796 | 44.11351777140203 |
| 0.5 LB + 0.5 mM CuCl2 | 50.162039054480054 | 49.837960945519946 |
| 0.5 LB + 2 mM formic acid | 51.811769102322614 | 48.188230897677386 |
| 0.5 LB + 2.5 mM malonic acid | 50.32897295454891 | 49.67102704545109 |
| 0.5 LB + 400 µM ZnCl2 | 53.38132877613711 | 46.61867122386289 |
| 0.5 LB + 4 mM malic acid | 53.61807968893593 | 46.38192031106408 |
