# Supplementary figures and images for: A novel short L-arginine responsive protein-coding gene (laoB) antiparallel overlapping to a CadC-like transcriptional regulator in Escherichia coli O157:H7 Sakai originated by overprinting
Source: BMC Evol Biol. 2018 Feb 12;18:21. doi: 10.1186/s12862-018-1134-0 (PMC5810103; doi:10.1186/s12862-018-1134-0)

## Slide 1
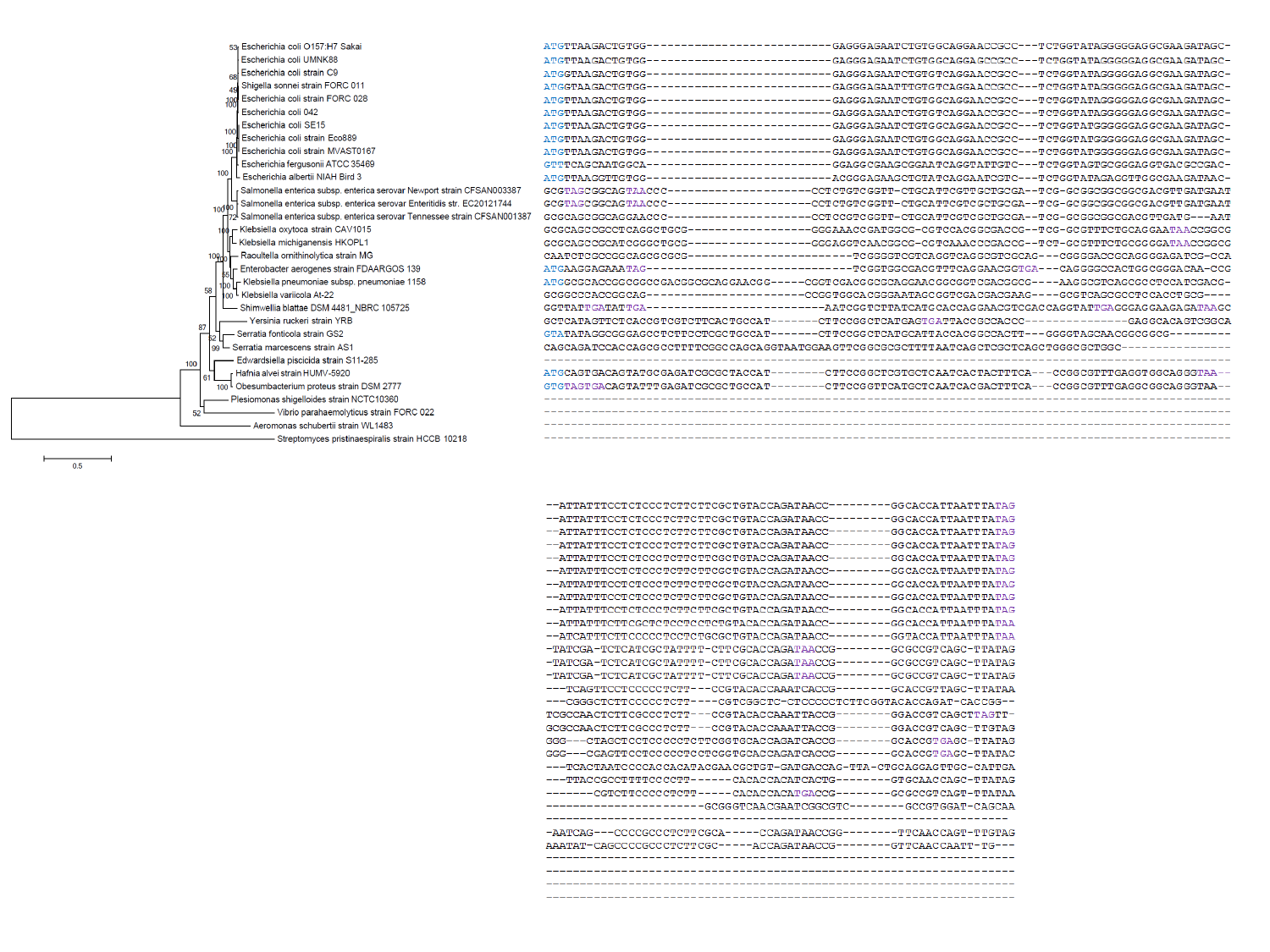

Supplement: Supplementary file 4 — Phylogenetic analysis of laoB at the DNA level. Start codons are colored in blue and stop codons in purple. (PPTX 1172 kb) [file 12862_2018_1134_MOESM4_ESM.pptx]

## Slide 1
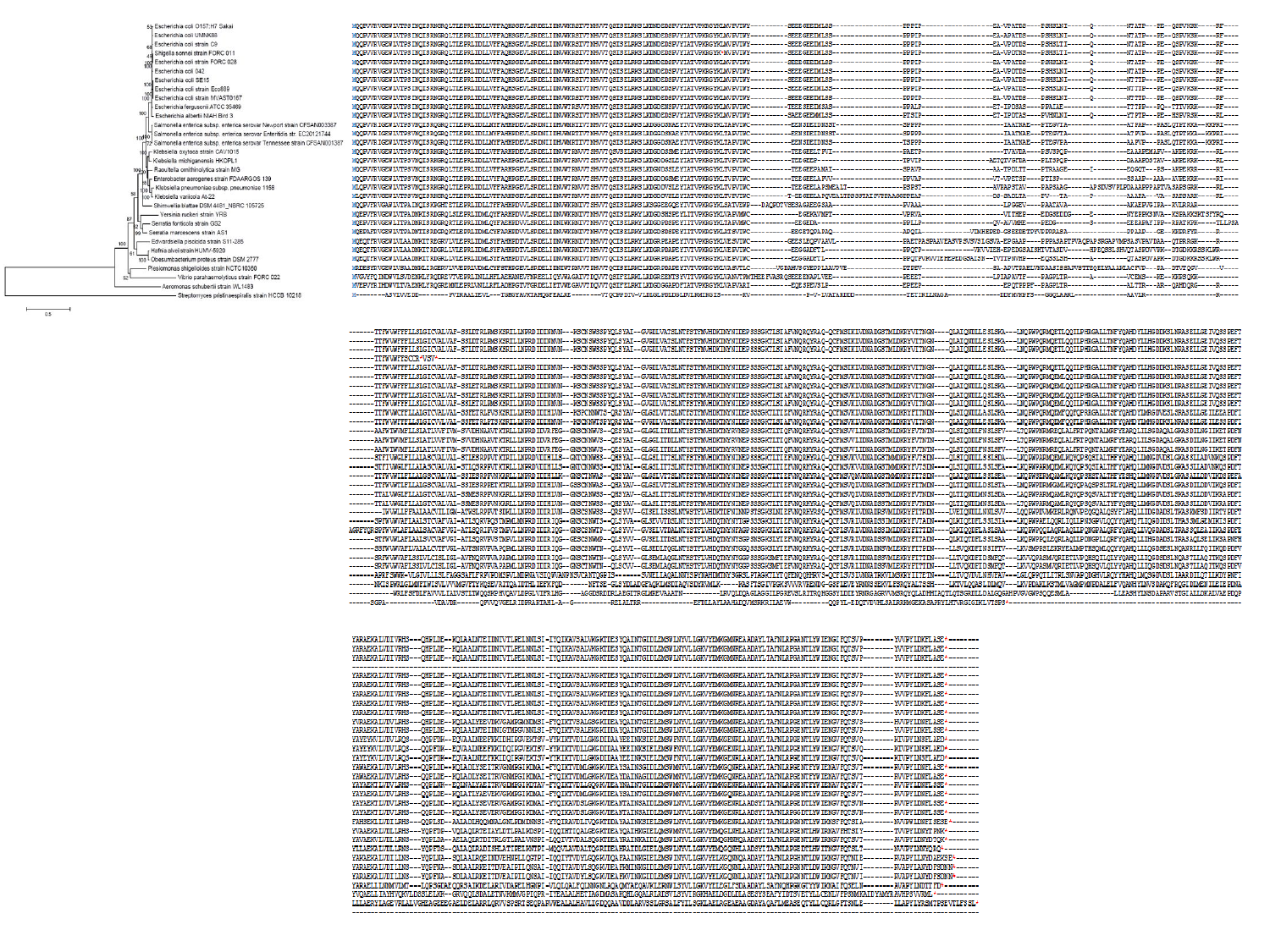

Supplement: Supplementary file 5 — Phylogenetic analysis of ECs5115 by the Maximum Likelihood method. The tree with the highest log likelihood (− 61,042.2643) is shown. The percentage of trees in which the associated taxa clustered together is shown next to the branches. Initial tree(s) for the heuristic search were obtained by applying the Neighbor-Joining method to a matrix of pairwise distances estimated using the Maximum Composite Likelihood (MCL) approach. A discrete Gamma distribution was used to model evolutionary rate differences among sites (5 categories; +G, parameter = 0.5123). The rate variation model allowed some sites to be evolutionarily invariable (+I, 32.8153% sites). The tree is drawn to scale, with branch lengths measured in the number of substitutions per site. The analysis involved 30 nucleotide sequences. All positions containing gaps and missing data were eliminated. There was a total of 8025 positions in the final dataset. Evolutionary analyses were conducted in MEGA6. In the alignment to the right, start codons are highlighted in blue and stop codons with a red asterisk. (PPTX 614 kb) [file 12862_2018_1134_MOESM5_ESM.pptx]
